# Boron Neutron Capture Therapy: A Review of Clinical Applications

**DOI:** 10.3389/fonc.2021.601820

**Published:** 2021-02-26

**Authors:** Timothy D. Malouff, Danushka S. Seneviratne, Daniel K. Ebner, William C. Stross, Mark R. Waddle, Daniel M. Trifiletti, Sunil Krishnan

**Affiliations:** ^1^ Department of Radiation Oncology, Mayo Clinic, Jacksonville, FL, United States; ^2^ Warren Alpert Medical School, Brown University, Providence, RI, United States

**Keywords:** boron neutron capture, fast neutrons, particles, radiation, boron neutron capture therapy (BNCT)

## Abstract

Boron neutron capture therapy (BNCT) is an emerging treatment modality aimed at improving the therapeutic ratio for traditionally difficult to treat tumors. BNCT utilizes boronated agents to preferentially deliver boron-10 to tumors, which, after undergoing irradiation with neutrons, yields litihium-7 and an alpha particle. The alpha particle has a short range, therefore preferentially affecting tumor tissues while sparing more distal normal tissues. To date, BNCT has been studied clinically in a variety of disease sites, including glioblastoma multiforme, meningioma, head and neck cancers, lung cancers, breast cancers, hepatocellular carcinoma, sarcomas, cutaneous malignancies, extramammary Paget’s disease, recurrent cancers, pediatric cancers, and metastatic disease. We aim to provide an up-to-date and comprehensive review of the studies of each of these disease sites, as well as a review on the challenges facing adoption of BNCT.

## Introduction 

Increasing the therapeutic ratio is one of the most significant challenges of modern clinical oncology. To this end, there has been significant interest in targeted therapies, with the goal of selectively treating tumor cells while sparing normal tissues. Boron neutron capture therapy (BNCT) is an emerging treatment modality aimed at improving the therapeutic ratio for traditionally difficult to treat tumors. BNCT was first proposed by Gordon Locher in 1936, who suggested that, if boron were able to be concentrated in the tumor and then exposed to thermal neutrons, the tumor would selectively receive a higher dose compared to normal tissues (1).

**Figure 1:**
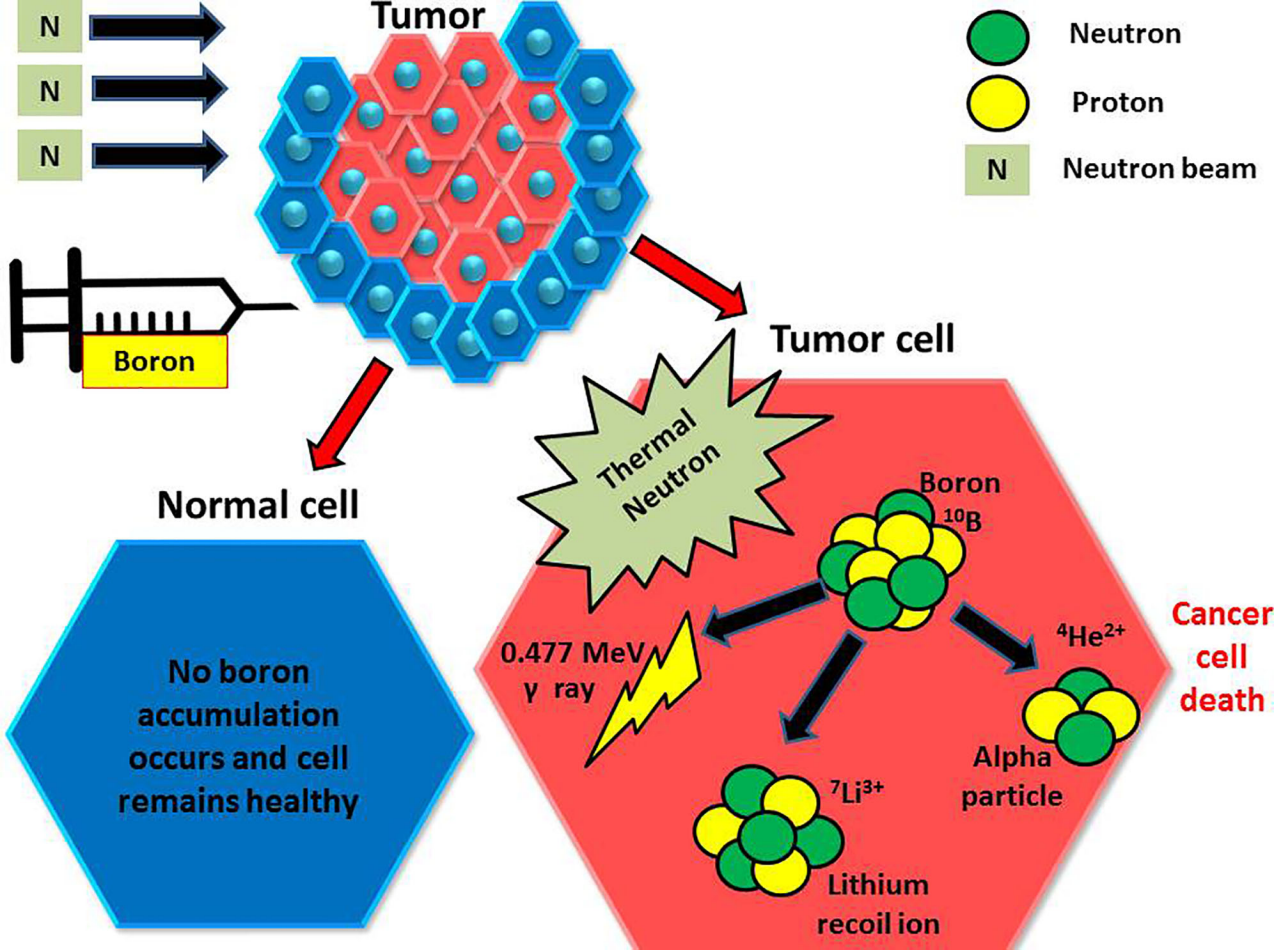
Injected boron compounds are preferentially found in tumor cells, which are then irradiated with thermal neutrons. The boron then undergoes a reaction, giving an alpha particle and an inert lithium ion. The alpha particle then damages the tumor cell with a finite range.

Treatment with BNCT is based on the nuclear capture and fission following the irradiation of nonradioactive boron-10 with low thermal neutrons (<0.025 eV), which leads to the production of an alpha particle and a recoiling lithium-7 (^10^B_5_ + ^1^n_0(th)_ → [^11^B_5_]* →^4^He_2_ (*α)*+^7^Li_3_ + 2.38 MeV). Alpha particles are a form of high linear energy transfer (LET) particles that deposit their energy over <10 μm, approximately the diameter of one cell (**Figure 1**) (1–3).

The most challenging aspect of successful treatment with BNCT is the delivery of boronated compounds to the tumor while avoiding significant uptake in normal tissues. The general requirements for successful boron delivery agents includes high tumor uptake, low normal tissue uptake, rapid clearance from tissue after treatment, and low toxicity (3). Boron delivery has been achieved typically with two agents: sodium borocaptate (BSH) and boronophenylalanine (BPA), with the latter complexed with fructose to form the more soluble BPA-F (2). The most efficacious boronated compound remains unclear, and trials have been variable as to which compound is given. The reader is referred to the text *Neutron Capture Therapy: Principles and Applications* for a more in-depth review of the technical aspects of treatment with BNCT (2).

In this review, our aim is to provide a comprehensive and updated summary of the current clinical literature for patients treated with BNCT. An overview of the largest studies is provided in **Table 1**.

**Table 1:** Summary of studies using BNCT by disease site.

Study	Number of patients	Boron carrier	Outcomes
**Glioblastoma Multiforme**			
Chanana et al. (4)	38	BPA-F	Median OS: 13 months Median time to progression: 31.6 months
Henriksson et al. (5)	30	BPA-F	Median OS: 14.2 months Median time to progression: 5.8 months
Kawabata et al. (6)	11	Combination BPA/BSH with EBRT	Median OS: 23.5 months
Kageji et al. (7)	23	BSH	Median survival: 19.5 months 5 year OS: 9.1%
Miyatake et al. (8)	167	BPA	Median OS: 10.8 months (recurrent) Median OS: 15.6 months (newly diagnosed)
**Head and Neck (Definitive)**			
Kankaanranta et al. (9)	30	BPA-F	Response rate: 76% Median PFS: 7.5 months 2 year OS: 30%
**Head and Neck (Recurrent)**			
Kato et al. (10)	26	BPA alone or BPA and BSH	Median survival: 13.6 months
Suzuki et al. (11)	62	BPA alone or BPA and BSH	Median survival: 10.1 months Response rate: 58% 2 year OS: 24.2%
Koivunoro et al. (12)	79	BPA-F	Complete response rate: 36% 2 year LRPFS 38% 2 year OS 21%
Wang et al. (13); Wang et al. (14)	23	BPA-F	2 year locoregional control: 28% 2 year OS: 47%
**Cutaneous Melanoma**			
Menendez et al. (15)	7	BPA	Response rate: 69%

BNCT, boron neutron capture therapy; BPA, boronophenylalanine; BSH, sodium borocaptate; EBRT, external beam radiation therapy; LRPFS, locoregional progression free survival; OS, overall survival; PFS, progression free survival.

## Glioblastoma Multiforme

Glioblastoma multiforme is one of the most challenging malignancies to treat, with a median survival of approximately 14 months despite maximum resection, radiation, and adjuvant chemotherapy (16). Due to this, BNCT has been proposed as a treatment option in the upfront and recurrent settings. Further, boron has been shown to possess direct tumoricidal activity and many can cross the blood-brain barrier (17). Interestingly, prolonged infusion of 6 h with BPA-F was found to have a survival advantage compared to 2 h infusions with similar toxicity (18).

Miyatake et al. reported on their experience treating 167 cases of malignant brain tumors and high grade meningiomas treated with BNCT from 2002 to 2014. In the recurrent setting, BPA was administered over a 2 h period at a dose of 200 mg/kg/h prior and 100 mg/kg/h during neutron irradiation. Epithermal neutrons were given, with a dose was chosen to keep the peak brain dose below 12.0 Gy equivalent. The median survival time for BNCT with BPA for recurrent GBM was 10.8 months and that for BNCT with BPA and BSH for newly diagnosed GBM was 15.6 months without an x-ray boost and 23.5 months with an x-ray boost. The biggest drawbacks to BNCT were radiation necrosis and symptomatic pseudoprogression (8). BNCT showed the most prominent survival benefit in recursive partitioning analysis (RPA) classes 3 and 7 (19).

In a series by Kawabata et al. seven patients received BNCT intraoperatively (sulfhydryl borane dose of 5 g/body) and eight patients received external beam BNCT (p-dihydroxyboryl-phenylalanine dose of 250 mg/kg) with epithermal neutrons as part for the treatment of newly diagnosed glioblastoma using sulfhydryl borane as the boron carrier. External beam BNCT was combined with photon therapy to a dose of 30 Gy in 15 fractions or 30.6 Gy in 17 fractions. The median time to progression for all patients was 11.9 months, with no difference between intraoperative (12.0 months) and external beam (11.9 months). The 2-year OS was 53.3%. Four patients developed grade 2 orbital edema, and one patient in the intraoperative arm developed grade 4 post-epileptic brain edema (20, 21).

Thirty patients with glioblastoma were treated between 2001 and 2003 (the pre-temozolomide era) with BNCT in Sweden. BPA-F at a high dose (900 mg/kg) was given as the boron carrier, with epithermal neutron irradiation 2 h after the infusion. The median OS was 14.2 months and the time to progression was 5.8 months. Seven patients experienced seizures, five experienced thromboembolic events, and eight had grades 1–3 skin toxicity. Quality of life was found to progressively deteriorate after BNCT (6).

In the United States, Chadha et al. reported on patients treated at Brookhaven National Laboratory in the mid-1990s designed to test the feasibility of single fraction BNCT with an epithermal neutron beam (22). In the past, when thermal neutron beams were used, patients underwent a craniotomy to allow the thermal beam to irradiate the tumor. Also, in this study, they performed a meticulous biodistribution analysis with tumor, blood, scalp, and normal brain (whenever possible) collected at the time of a second debulking craniotomy and noted that tumor concentration was about 3.5 times that in blood, scalp concentration was about 1.5 times that in blood, and normal brain concentration was less than that in blood. BNCT was performed about 4 weeks after the surgery with a repeat dose of BPA-F and the prescription dose was based on normal brain tolerance with normal brain boron concentration assumed to be that of blood to account for brain endothelial dose. The median OS, albeit in just 10 patients, was 13.5 months at a time when the OS in cooperative group trials was 9.7 months. A total of 38 patients with glioblastoma were treated with BPA-fructose at a dose of 250 or 290 mg/kg as part of the phase I/II dose-escalation study. The median time to tumor progression was 31.6 weeks, with a median survival of 13.0 months. There were no grade 3 or 4 toxicities (4).

Kageji et al. reported on 23 newly diagnosed GBM patients treated with BNCT and without additional chemotherapy. 100 mg/kg BSH was given and patients underwent craniotomy for direct delivery of thermal neutrons to the tumor. The median survival was 19.5 months, with 2- and 5-year survivals of 31.8% and 9.1%, respectively. Toxicity was not reported (7, 23). Of note, five patients treated with BNCT survived more than 3 years after diagnosis (24). Notably, there are long term survivors treated with BNCT for high grade gliomas. In one series from 1994, of the 120 patients treated with BNCT for brain tumors, there were nine patients who had survivals of more than 10 years (25).

In the setting of recurrent disease, a phase I study by Kankaanranta et al. investigated the use of L-BPA-fructose in increasing doses ranging from 290 mg/kg to 450 mg/kg for patients with glioblastoma or anaplastic astrocytoma that progressed more than 6 months after surgery and external beam radiation therapy. The median survival following BNCT was 7 months. Four of the six patients at the 450 mg/kg dose level experienced grade 3 adverse effects, the most frequent being seizures. On subset analysis, patients who received >290 mg/kg L-BPA-fructose or those who received >34 Gy weighted dose to their planning target volumes had better outcomes than those who received 290 mg/kg L-BPA-fructose or ≤34 Gy weighted dose to their PTVs, respectively. The authors concluded that L-BPA-fructose at a dose of 400 mg/kg as a 2-h infusion is reasonable for recurrent gliomas (26). Aiyama et al. reported on a 54-year-old male with recurrent GBM and a 64-year-old female with atypical meningioma treated with BNC T in Japan. In the two cases, the only adverse event was grade 2 conjunctivitis, and the authors concluded that BNCT is effective and safe as palliative therapy for malignant brain tumors (27). Okazaki et al. reported on a 22-year-old patient with GBM who developed a total of three recurrences. BNCT was delivered using a dose of 100 mg/kg BSH. The patient survived for 9 years following BNCT, but ultimately developed carcinomatous encephalomyelopathy (28).

Given the increased use of protons, there has been interest in combining proton radiotherapy with BNCT. Patients receiving BNCT with proton therapy showed a better survival than those receiving radiation and temozolomide in a small study, although this was not statistically significant (29). Further research is needed to determine if there is a benefit to combination proton therapy and BNCT.

One of the concerns of BNCT in the setting of malignant gliomas is the high rates of symptomatic pseudoprogression and radionecrosis (30). In one series, 11 out of 52 malignant glioma and three out of 13 malignant meningioma patients developed increases in edema following BNCT at 3 months (31). Due to the rates of radionecrosis following BNCT, researchers in Japan developed a pilot study for BNCT using BPA in combination with bevacizumab. Bevacizumab was started 2–6 weeks after BNCT and was given in 10 mg/kg doses biweekly. From 2013 to 2014, seven patients were treated with BNCT and bevacizumab. Median OS and PFS were 15.1 months and 5.4 months, respectively. There was one death due to uncontrolled edema after bevacizumab was interrupted due to meningitis. No radionecrosis was seen through December 2017, and the authors concluded that bevacizumab treatments prevented radionecrosis with prolonged OS and acceptable toxicity (32). Moreover, bevacizumab at a dose of 5 mg/kg was shown to improve symptomatic pseudoprogression in two patients treated with BNCT for recurrent gliomas. The authors concluded that BNCT in combination with bevacizumab may prolong survival (33).

In support of this, Miyatake et al. reported on four patients with recurrent malignant gliomas treated in Osaka, Japan with BNCT and bevacizumab. Of the three patients with RPA class 3, the survival time after BNCT was 14, 16.5, and over 23 months. The patient with an RPA class of 4 survived over 26 months. The authors concluded that BNCT with bevacizumab improved symptoms of symptomatic pseudoprogression or radionecrosis and prolonged survival (34).

Histopathological studies were performed on eight patients treated with BNCT for GBM either at the time of salvage surgery or autopsy. Tissue studies demonstrated residual tumor cells in four patients. The authors concluded that a dose of 68 Gy to the GTV and 44 Gy to the CTV was needed for histopathologic cure (35). In one interesting case of a patient with gliosarcoma previously treated with BNCT, only the sarcomatous component recurred 6 months post-BNCT (36). Radiographically, 50% of patients had cerebral changes within the first year of treatment, with atrophy affecting 42% of patients analyzed as part of the EORTC 11961 trial (37).

There are several current trials investigating the use of BNCT in high grade gliomas. In Japan, 21 patients with newly diagnosed glioblastoma were treated with combination BPA and BSH. Protocol 1 investigated 10 patients treated with BNCT alone with Protocol 2 investigating 11 patients receiving external beam radiation therapy. The median survival time was 15.6 months for all patients, and 23.5 months for Protocol 2. The 2-year overall survival was 25% (1, 6, 20). A phase II clinical trial (OSAKA-TRIBRAIN0902, NCT00974987) was designed based on that study and completed accrual in 2018. The Tsukuba BNCT trial is a phase II study evaluating combined photon irradiation with concurrent temozolomide combined with BNCT using 250 mg/kg BPA (38).

## Meningioma

Miyatake et al. described seven cases of malignant meningiomas, including three anaplastic meningiomas, two papillary meningiomas, one atypical meningioma, and one sarcoma transformed from a meningioma, treated with BNCT. 18F-BPA PET was applied before BNCT in six patients and one underwent methionine-PET. Two of the three anaplastic meningiomas showed a complete response, and all six patients analyzed showing radiographic improvement (39). Stenstam et al. described two patients treated with BNCT using BPA-fructose (900 mg/kg body weight) for recurrent meningeal tumors following surgery, radiation, and salvage surgery and concluded that BNCT is a potentially effective modality for malignant intracranial meningeal tumors (40).

Similarly, the median survival times after BNCT with BPA for high grade meningiomas recurrent after or refractory to treatment was 14.1 months from BNCT treatment in the study by Kawabata et al. In this study, BPA was administered prior to neutron irradiation (200 mg/kg/h) and during neutron irradiation (100 mg/kg/h). The duration was determined to not exceed the dose of 15 Gy-Eq to the normal brain (7, 41). Of 20 patients who underwent 28 BNCT treatments following at least one prior course of external beam radiation therapy or stereotactic radiosurgery, at least three patients had pseudoprogression, with five patients experiencing symptomatic radiation necrosis (41).

A retrospective review from the Osaka Medical College Hospital and the Kyoto University Research Reactor Institute investigated 31 patients treated with BNCT for recurrent high grade meningiomas, including seven skull base meningiomas. PET scans revealed a 3.8 times higher boron accumulation in the meningiomas compared to normal brain, with a mean maximum absorbed dose of 67.2 Gy-Eq. All lesions showed decrease in size, and the median survival of skull base meningiomas following BNCT was 24.6 months (42).

Tamura et al. reported on a 25-year-old patient with a recurrent malignant meningioma who underwent two resections and three courses of Gamma Knife radiosurgery without adequate control. She received 5 g BSH IV for 1 h approximately 13 h before radiation, and 500 mg/kg BPA before receiving epithermal neutrons. The minimum tumor dose was estimated to be 39.7 Gy-Eq. She regained the ability to ambulate within 1 week after the first treatment of BNCT and showed decrease in size of the tumor at 26 weeks (43).

A pathology study of a 70-year-old who died from systemic metastasis from anaplastic meningioma showed significantly lower proliferative activity of the meningioma recurrence compared to an untreated metastatic liver lesion and untreated meningioma. The study supports the early effect of BNCT on anaplastic meningiomas, with treatment effect seen as early as 2.5 months after treatment (44). Other pathology studies showed that radiation-induced focal venular fibrinoid necrosis and multifocal demyelination may occur after high doses of BNCT to neuroparenchyma (45).

## Definitive Treatment for Head and Neck Cancers

Although the majority of clinical trials regarding BNCT use in head and neck malignancies investigated it in the recurrent setting, BNCT has been used for definitive therapy as well. Single fraction BNCT using BPA-F at a dose of 400 mg/kg with epithermal neutrons using two circular 14 cm diameter beams with irradiation times of 15.3 min and 16.5 min has been used successfully for the treatment of unresectable, undifferentiated sinonasal carcinoma. The authors reported that although the patient recurred 6 months post-treatment, his quality of life improved following treatment and primary side effect experienced was mucositis (46). Kimura et al. reported on a 78-year-old patient with a papillary cystadenocarcinoma of the upper lip treated with BNCT using 500 mg/kg BPA as the boron carrier in two fractions with a total dose 63.4 Gy-Eq at the tumor peak. The tumor decreased by 86% at 5-month follow up, although the patient experienced acute extensive erosion (47).

Recently, Kankaanranta et al. reported on a 53-year-old woman successfully treated with BNCT for a large head and neck cancer in the definitive setting. The patient presented with a 7.4 cm intranasal mass and was treated with 400 mg/kg L-BPA-fructose followed by IMRT to a dose of 44 Gy following resolution of acute BNCT induced mucositis. Intravenous cetuximab and cisplatin were given concurrently with IMRT. The patient experienced grade 3 mucositis, alopecia, fatigue, and xerophthalmia. The patient achieved a complete response and had no evidence of disease at 6 month follow-up (48). The authors concluded that BNCT is a reasonable treatment with moderate toxicity in the setting of first line therapy for head and neck cancers.

Kankaanranta et al. reported on a prospective, phase I/II trial of 30 patients treated for inoperable, locally advanced head and neck cancer with BNCT between December 2003 and September 2008 in Finland (NCT00114790). Of the 30 patients, 29 had carcinomas as the primary histology, with one patient diagnosed with a sarcoma. Patients were treated with surgery and radiation therapy to a median dose of 60 Gy, with 33% of patients receiving concurrent chemotherapy. BNCT was administered in two fractions with 400 mg/kg L-BPA-fructose with neutrons given from two portals with a median beam time of 18.6 min. Of the 29 evaluable patients, there was a 76% response rate. The median PFS was 7.5 months, with a 2-year OS and PFS of 30% and 20%, respectively. Acute grade 3 mucositis and oral pain were noted in 54% of patients, with fatigue in 32% of patients. Three patients developed grade 3 osteoradionecrosis and one patient developed grade 4 soft tissue necrosis. Twenty percent of patients developed late grade 3 xerostomia (9).

Fatal carotid blowout remains a concern following BNCT for head and neck cancers, with one study by Aihara et al. reporting carotid blowout syndrome in two out of 33 patients treated with BNCT, developing between 1 and 3 months after BNCT (49).

## Recurrent Head and Neck Cancers

Kato et al. reported on the first six patients treated for recurrent head and neck cancers with BNCT, using combination BPA (250 mg/kg) and BSH (5 g) with epithermal neutron irradiation with a fluence ranging from 1.3 to 2.7. An improvement in quality of life was seen in five patients given the reduction in tumor volume (50). Suzuki et al. retrospectively reviewed the records of patients treated for locally recurrent or unresectable head and neck cancers treated with BNCT between 2001 and 2007 at Kyoto University. BPA alone or BPA and BSH were used as the boron compounds. For the 62 patients treated, the median follow up was 18.7 months, the median survival was 10.1 months, the overall response rate was 58% at 6 months, and the 2-year OS was 24.2%. Hyperamylasemia was the most common acute grade 3 or 4 toxicity (38.6%), followed by mucositis (9.7%) and pain (9.7%). Two patients had fatal carotid hemorrhage, and one patient died due to malnutrition (11). In another retrospective study, 79 patients with inoperable, locally recurrent squamous cell carcinoma of the head and neck were treated with BNCT in Finland between 2003 and 2012. Ninety-five percent of patients had previously received radiation to a median dose of 66 Gy. L-BPA-fructose was used at a dose of 350–400 mg/kg, with neutron irradiation lasting a median of 42 min. Thirty-nine patients received BNCT twice, while 40 patients received one fraction due to a variety of reasons, such as distant disease or medical comorbidities. Four of this patient cohort, 68% showed some response, with a 36% complete response rate. Patients treated twice with BNCT showed improved response compared to those who were treated once. With a median follow-up of 7.8 years, the 2-year locoregional progression free survival was 38% and 2-year OS was 21%. A minimum GTV dose of 18 Gy was associated with the best survival, suggesting that minimum tumor dose is predictive of survival (12).

Based on 26 patients treated with recurrent head and neck malignancies in Osaka, Japan since 2001, Kato et al. found an overall response rate of 85%, with improvement in quality of life. Combination BSH and BPA, or BPA alone (250 or 500 mg/kg) were used. The mean survival following treatment was 13.6 months. Transient mucositis and alopecia were the most common adverse effects, with three patients developing osteomyelitis and one suffering from brain necrosis. The authors conclude that BNCT is a new and promising technique (10). Another study reviewed 12 patients with inoperable, recurred, locally advanced head and neck cancers. L-BPA-F was given at a dose of 400 mg/kg followed by neutron irradiation, with the median time from the first field of 18.1 min and time of irradiation from the second field of 17.5 min. Eighty-three percent of patients had a response to BNCT; with 33% of patients without recurrence at a median follow up of 1.0 months. Two patients had grade 3 toxicity: one patient experienced xerostomia and one experienced dysphagia (51). Aihara et al. reported on 10 patients treated with recurrent squamous cell carcinoma and seven patients with recurrent and three newly diagnosed head and neck non-squamous cell carcinoma treated between 2003 and 2007 in Japan. Of these, 11 patients showed a complete response, with a total response of 90%. There were no severe acute or late toxicities (52).

In Finland, six patients with locally recurrent laryngeal squamous cell carcinoma and three patients with persistent laryngeal cancer were treated with BNCT from 2006 to 2012. L-BPA-F at a dose of 400 mg/kg was administered over 2 h. Of the eight patients analyzed, there were two complete responses and four partial responses. Five patients developed early large grade 3 toxicity and 38% developed late grade 3 toxicity. The most common acute and late toxicities were stomatitis and mucositis. The median time to progression was 6.6 months (53).

Haginomori et al. reported on the first case of a 42-year-old patient treated for extensive squamous cell carcinoma of the temporal bone that recurred after initial chemotherapy, surgery, and radiation therapy. The patient underwent planned fractionated BNCT with BPA with two treatments given 1 month apart. The total radiation dose to the deepest point of the tumor was approximately 36.9 Gy-Eq. At 6 months after the first treatment, there was no evidence of residual tumor (1, 54).

In recurrent salivary gland cancers, a 48-year-old patient with recurrent submandibular gland malignancy undergoing 18F-BPA PET before BNCT showed complete regression after therapy (55). Aihara et al. reported on two patients with recurrent salivary gland cancer and three patients with newly diagnosed T4 salivary gland cancers treated with BNCT between 2003 and 2007. All patients achieved a complete response within 6 months. The median survival was 32 months, with two patients with distant metastatic disease. There were no severe grade 3 or high toxicity (56).

BNCT has also been used successfully in treating nodal recurrences. Four patients were enrolled at Osaka Medical College evaluating the use of BNCT for regional nodal recurrence of oral cavity cancers. All patients showed a partial response, with one patient having a marked improvement in quality of life, following administration of 500 mg/kg BPA. The neutron dose was determined by delivering 10–15 Gy-Eq to the oral mucosa (57). Of six patients treated at the same institution for recurrent oral cancer, three remained alive with improvement in quality of life. Five patients had decrease in pain (58), suggesting that BNCT may be beneficial for palliation.

Using the Tsing-Hua Open Pool Reactor (THOR) at the National Tsing-Hua University in Hsin-Chu, researchers initially enrolled 17 patients with 23 recurrent head and neck tumors between 2010 and 2013 in a phase I/II trial investigating BNCT for recurrent head and neck cancers. A fructose complex of L- BPA was used. Patients were then treated to a prescription dose of 20 Gy-Eq to cover 80% of the gross tumor using a single field and were treated in two fractions at 28-day intervals. With a median follow up of 19.9 months, 15 patients received both fractions, and six had a complete response. Nine patients reported improved quality of life, with low-grade oral mucositis, radiation dermatitis, and alopecia as the most common acute toxicities. One patient developed grade 4 acute laryngeal edema and carotid hemorrhage, and two patients developed late grade 3 cranial neuropathy. The 2-year overall survival was 47% (13, 14, 59). The 2-year locoregional control rate was 28%, and a second trial using image-guided IMRT was initiated in 2014 to improve local control. In this second protocol, IMRT was initiated 28 days after one administration of BNCT. Of the seven patients treated with this protocol, three had a complete response with a 1-year OS of 56%. Toxicity was similar to the first trial, with one patient developing grade 4 oral bleeding and another developing grade 4 dyspnea due to facial edema (14). Using nine patients with recurrent head and neck cancer from THOR, Lee et al. analyzed the dose distributions between BNCT alone and BNCT with IMRT. BNCT with IMRT had GTV conformity and improved homogeneity compared to BNCT monotherapy (60).

Eight patients underwent BNCT with IV BPA and seven patients were treated with intra-arterial BPA for recurrent head and neck malignancies. Efficacy was similar, and the authors determined that intra-arterial BPA is a viable delivery system for BNCT (61).

## Lung Cancers

BNCT has been proposed for diffuse, non-resectable lung tumors (62), as well as for inoperable malignant pleural mesothelioma (63–65). Suzuki et al. reported on two patients with diffuse pleural tumors, one with malignant pleural mesothelioma and one with malignant short spindle cell tumor, treated with BNCT with BPA-F to a dose of 250 or 500 mg/kg. The tumors either were stable or regressed at 6-month follow up with no grade 3 or higher acute or late toxicities (63). The feasibility of treating shallow lung tumors with BNCT was confirmed in one study, although the role of BNCT in treating deeper tumors remains unknown (66).

## Breast Cancers

There have been few studies to date investigating the use of BNCT in breast malignancies, although BNCT may have a role as a potential option for treating HER2 overexpressing breast cancers based on promising pre-clinical data. Immunoliposomes, such as those labeled with trastuzumab, have been proposed to act as a boron carrier and can selectively target HER2 overexpressing cells (67). Further, dosimetric analyses have shown the possibility of BNCT for locally recurrent breast cancer (68). Collectively, these studies suggest a benefit of BNCT for breast cancers, but further clinical studies are needed.

## Hepatocellular Carcinoma

Suzuki et al. reported on the first patient treated for multiple hepatocellular carcinomas in Japan. The patient had Child-Pugh grade B cirrhosis, and irradiation was confined to the right lobe. BPA (250 mg/kg) and BSA (1 g/body) were used as boron carriers. The peak dose to the right lobe of the liver was 4.9 Gy-Eq, and the mean dose was 2.7 Gy-Eq. At 1 month, the tumors treated with BNCT remained stable, although there was progression of disease after 3.5 months (69).

Yangie et al. performed a pilot study using selective intra-arterial infusion to deliver a BSH containing water-in-oil-in-water emulsion to a left liver lobe lesion in a 63-year-old man with hepatocellular carcinoma. Irradiation time was set to limit the maximum dose to the liver of 5.0 Gy-Eq. The patient was considered to have stable disease on initial follow-up imaging, although he later developed multiple nodules in the left lobe of the liver as well as lung metastatic disease. The patient died from pneumonia 7 months after BNCT (70).

## Sarcomas

Osteosarcoma has been shown to be effectively and safely treated with BNCT. BNCT has also been used successfully in the treatment of osteosarcoma of the temporomandibular joint, with no evidence of recurrence after approximately 2 years (71). Futamura et al. reported on a 54-year-old female effectively treated for a recurrent radiation-induced osteosarcoma in the left occipital skull by BNCT. 500 mg/kg of BPA was administered. Although she was unable to ambulate at diagnosis, she regained the ability to ambulate without aid approximately 3 weeks following BNCT. The treatment was well tolerated, with the patient experiencing alopecia as the only reported toxicity (72).

Malignant peripheral nerve sheath tumors (MPNSTs) are a rare soft tissue malignancy with a poor prognosis despite surgical resection. Animal models using L-BPA showed efficacy of BNCT for MPNST (73), and a 70-year-old woman were treated for a MPNST in the right supraclavicular fossa with an initial response and no evidence of recurrence at 2 years (74). Animal models were promising for clear cell carcinoma (75).

## Cutaneous Melanoma

Patients diagnosed with melanoma often have poor prognoses despite optimal treatments. Due to this, Gonzalez et al. reported on a case of a 54-year-old women treated for cutaneous melanoma as part of the initial 30 patient cohort treated with BNCT. BPA-F to a dose of 14 g/m2 over 90 min was used with an estimated treatment time of 903 monitor units to keep the normal maximum skin dose below 16.5 RBEGy. Of the 25 skin nodules, 21 were in complete response 8 weeks following treatment with grade 1 acute skin reaction as the primary toxicity (76).

Further, Menendez et al. reported on seven patients treated with BNCT for cutaneous melanoma with multiple skin metastases in the extremities in Argentina between 2003 and 2007. All patients received 14 mg/m2 of BPA. The overall response rate was 69%, with a 30% grade 3 toxicity rate (ulceration) (15). Two patients enrolled in the trial were studied using dynamic infrared imaging, which registers the temperature evolution of normal skin and tumor. Researchers found the main erythematous reaction occurred between the second and 5th week after irradiation (77).

Most recently, the Third Xiangya Hospital of Central South University in Changsha China recently developed a protocol for treating malignant melanoma using BNCT, with a goal of accrual of 30 patients (NCT02759536). The authors report on the first patient treated for a left foot lesion in August 2014. In this study, BPA-F complex was used with 350 mg/kg infused into the patient over 90 min. Using the Monte Carlo N Particle Transport Code 6 program, the estimated dose was determined, and the patient was treated with two fields in a total of 20 min. The patient experienced only mild dandruff `1 week following irradiation, although this progressed to grade 2 dermatitis at 4 weeks. There were no significant lab value findings. Biopsy performed at 9 months post-BNCT and PET scan 24 months post-BNCT showed no evidence of disease (78). BNCT may also be used in controlling in-transit and lymph node metastasis from cutaneous melanoma (79).

## Extramammary Paget’s Disease

Due to the morbidity associated with wide local excision of extramammary Paget’s disease of the genitals, Makino et al. reported on the first two cases of extramammary Paget’s disease of the genitals treated with BNCT. Both patients were over the age of 70. At 12 months after treatment, both cases had a complete response with no evidence of recurrence or metastatic disease (1, 80).

Kyoto University treated one patient with vulvar melanoma and three with genital extramammary Paget’s disease between 2005 and 2014. ^10^B-enriched L-BPA was used as the boron delivery source to a dose of 200 mg/kg over 3 h (rate of 80 mg/kg/h for the first 2 h and 40 mg/kg/h for the last hour). Patients were irradiated in the last hour of the infusion. All four patients had a complete response in 6 months, with two patients developing grade 2 erosions, one patient developing grade 2 dysuria, and one patient developing grade 1 mucositis (81).

## Metastatic Disease

Although currently limited to translational studies and case reports, BNCT will likely be used in the setting of metastatic disease. The EORTC 11001 protocol is a translational phase I trial with the goal to measure the uptake of two boronated compounds in tissues and the blood. BSH and BPA are administered prior to surgical resection of hepatic metastasis, with no patients experiencing toxicity from the boron carriers. BSH was not a suitable carrier, as the liver concentration was higher than in the metastasis. BPA may be used for extracorporeal irradiation of the liver with BNCT (82), which has previously been used in a small series of two patients (83). The study was also performed for head and neck cancer patients, and found that BPA and BSH might allow effective treatment in squamous cell carcinoma (84). The study was repeated in thyroid cancer (85).

Interestingly, a proof of principle study using rats treated with BNCT showed that BNCT is capable of inducing the abscopal effect in rats inoculated with colon cancer cells (86). Further studies are needed to evaluate the immunogenicity of BNCT.

Clinically, BNCT has been shown to lead to suppression of tumor growth for 2 months in a 72-year-old man treated with recurrent gastric cancer and a left cervical node lesion (1, 87).

## Pediatrics

BNCT has also shown benefit in children with malignant brain tumors. In one series, 23 patients under the age of 15 treated with BNCT were included. Four patients were under the age of 3. Three patients had glioblastomas, six patients with anaplastic astrocytomas, seven patients with PNET tumors, six patients with pontine gliomas, and one patient with anaplastic ependymoma. Four of the six anaplastic astrocytoma and the anaplastic ependymoma patients had no evidence of recurrence. The patients with GBM and PNET tumors died of disseminated tumor without local recurrence. The pontine glioma patients died of tumor regrowth. The authors concluded that BNCT can be used in children (88).

Zhang et al. analyzed the secondary malignancy risk in pediatric patients treated with BNCT for brain tumors in China. When comparing neutron beam geometries, the authors concluded that the lifetime attributable risk of secondary malignancy was lower with posterior-to-anterior arrangement compared to right-lateral and top-to-bottom. Younger patients and female patients also had higher risks of secondary malignancy (89). In Japan, only 1 out of 180 patients treated for malignant brain tumors since 1968 developed multiple radiation-induced meningiomas in the treatment field (90).

## Discussion

BNCT represents a promising treatment modality, with data suggesting the safety and efficacy of treatment in patients with advanced tumors. Caution should be taken when interpreting the data from BNCT. The studies exhibit a high degree of heterogeneity in inclusion criteria, boronated compounds used, times for infusion, and neutron dose given, which creates difficulty in comparing studies even within the same disease site. Additionally, there are no current studies investigating the outcomes of BNCT compared to other standards of care, which limit interpretation of the results. Well-designed phase II/III studies are needed to define the efficacy and safety of BNCT in a variety of tumor types.

Although initial results with BNCT are promising, toxicity rates remains relatively high. Further research into developing more selective boronated compounds is needed to improve the therapeutic ratio of treatment and decrease potential toxicity. In the era of immunotherapy and targeted agents, ^10^B can conceivably be conjugated to these agents to increase selectivity, an area of needed research. Alternatively, BNCT may be coupled with immunotherapy to achieve optimum synergy between immune activation by the high LET attributes of BNCT and immunotherapy that maintains lymphocytes in an activated state.

Another barrier to adoption of BNCT is the high cost of developing and maintaining a BNCT treatment center. Currently, there are no centers in the United States treating with BNCT. Nakagawa et al. estimated that a BNCT facility in Japan costs approximately 1200 million Yen (approximately $11.4 million) to construct with an annual personnel cost of 113 million Yen (approximately $1 million) (91). The substantial initial startup costs are a barrier to developing a BNCT center in the United States, especially with a lack of studies investigating the cost effectiveness of BNCT compared to other modalities.

Another potential area of research is using non-boronated compounds, which, while offering similar mechanisms as BNCT, may have improved treatment effects or mitigation of BNCT limitations. ^157^Gadolinium in particular has generated considerable interest, in no small part due to its role as a contrast agent in MRI and notable high uptake in (brain) tumor cells, where the large magnetic moment of the Gd3+ ion may be detected (92–96). Unlike ^10^B, which releases both high LET He and Li ions, the ^157^Gd (n,γ)^158^Gd capture reaction generates gamma rays, x-rays, and internal conversion, Auger, and Coster-Kronig electrons. These electrons are similarly high-LET with limited range, concentrating damage within a diameter of approximately one cell and effectively generating double strand breaks. This offers unique potential if a highly localizing gadolinium-based agent can be achieved. However, the presence of gamma and x-rays broadens the dose delivery region, and may somewhat limit selectivity (94). In comparison with boronated compounds, the wider irradiation range may offer improved treatment of nearby cells undergoing limited deposition, forming a spectrum of utility amongst boronated and gadolinium-based agents. As such, treatment efforts hinge on the development of gadolinium-based compounds, of which many have been developed and assayed (94), though deployment within *in vivo* models has been limited. Early results were promising, such as Tokumitsu and colleagues’ deployment of chitosan nanoparticles in 1999, finding significant suppression in a B16F10 murine melanoma model (95). Uniquely, combination agents have been introduced: a gadolinium/boron agent, bound to low-density lipoproteins (LDL), has demonstrated preliminary success in MRI-monitorable detection and treatment success both in-vitro and in a murine model (93).

## Conclusions

Boron neutron capture therapy represents an emerging targeted therapy with promising results and acceptable toxicity in early clinical studies. Further prospective research is necessary to define the role of BNCT in clinical practice.

## Author Contributions

TM, DT, and SK were responsible for the concept of the manuscript. All authors contributed to the article and approved the submitted version.

## Conflict of Interest

DT has received clinical trial funding from Novocure and publishing fees from Springer.

The remaining authors declare that the research was conducted in the absence of any commercial or financial relationships that could be construed as a potential conflict of interest.
